# Ultrastructural and glycoproteomic characterization of *Prevotella intermedia*: Insights into *O*‐glycosylation and outer membrane vesicles

**DOI:** 10.1002/mbo3.1401

**Published:** 2024-02-26

**Authors:** Xi Ye, Bindusmita Paul, Joyce Mo, Eric C. Reynolds, Debnath Ghosal, Paul D. Veith

**Affiliations:** ^1^ Department of Biochemistry and Pharmacology, Bio21 Molecular Science and Biotechnology Institute The University of Melbourne Melbourne Victoria Australia; ^2^ Oral Health Cooperative Research Centre, Melbourne Dental School, Bio21 Institute The University of Melbourne Parkville Victoria Australia; ^3^ ARC Centre for Cryo‐electron Microscopy of Membrane Proteins, Bio21 Molecular Science and Biotechnology Institute University of Melbourne Parkville Victoria Australia

**Keywords:** electron microscopy, *O*‐glycosylation, outer membrane vesicles, *Prevotella intermedia*, proteome, tomography

## Abstract

*Prevotella intermedia*, a Gram‐negative bacterium from the Bacteroidota phylum, is associated with periodontitis. Other species within this phylum are known to possess the general *O*‐glycosylation system. The *O*‐glycoproteome has been characterized in several species, including *Tannerella forsythia*, *Porphyromonas gingivalis*, and *Flavobacterium johnsoniae*. In our study, we used electron cryotomography (cryoET) and glycoproteomics to reveal the ultrastructure of *P. intermedia* and characterize its *O*‐glycoproteome. Our cryoET analysis unveiled the ultrastructural details of the cell envelope and outer membrane vesicles (OMVs) of *P. intermedia*. We observed an electron‐dense surface layer surrounding both cells and OMVs. The OMVs were often large (>200 nm) and presented two types, with lumens being either electron‐dense or translucent. LC‐MS/MS analyses of *P. intermedia* fractions led to the identification of 1655 proteins, which included 62 predicted T9SS cargo proteins. Within the glycoproteome, we identified 443 unique *O*‐glycosylation sites within 224 glycoproteins. Interestingly, the *O*‐glycosylation motif exhibited a broader range than reported in other species, with *O*‐glycosylation found at D(S/T)(A/I/L/M/T/V/S/C/G/F/N/E/Q/D/P). We identified a single *O*‐glycan with a delta mass of 1531.48 Da. Its sequence was determined by MS2 and MS3 analyses using both collision‐induced dissociation and high‐energy collisional dissociation fragmentation modes. After partial deglycosylation with trifluoromethanesulfonic acid, the *O*‐glycan sequence was confirmed to be dHex‐dHex‐HexNAc (HPO_3_‐C_6_H_12_O_5_)‐dHex‐Hex‐HexA‐Hex(dHex). Bioinformatic analyses predicted the localization of *O*‐glycoproteins, with 73 periplasmic proteins, 53 inner membrane proteins, 52 lipoproteins, 26 outer membrane proteins, and 14 proteins secreted by the T9SS.

## Graphical abstract

Electron cryotomography analysis revealed ultrastructural details of *Prevotella intermedia*, revealing an electron‐dense surface layer surrounding both cells and outer membrane vesicles (OMVs). Mass spectrometry analysis enabled the identification of a single major *O*‐glycan [dHex‐dHex‐HexNAc(HPO_3_‐C_6_H_12_O_5_)‐dHex‐Hex‐HexA‐Hex(dHex)] found in 443 unique sites within 224 glycoproteins. Bioinformatic analyses of *O*‐glycoprotein localization predicted 73 periplasmic proteins, 53 inner membrane proteins, 52 lipoproteins, 26 outer membrane proteins, and 14 proteins secreted by the type IX secretion system.

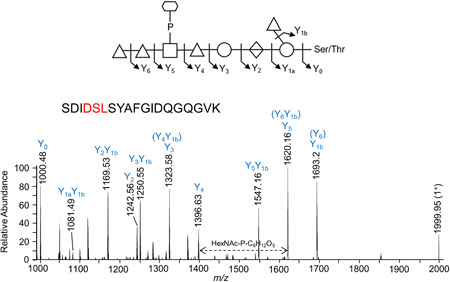

## 1 INTRODUCTION

*Prevotella intermedia* is a Gram‐negative, black‐pigmented, nonmotile, rod‐shaped bacterium. It predominantly colonizes subgingival regions as an obligate anaerobe and is notably linked with periodontitis. Periodontitis is a chronic inflammatory disease primarily due to the accumulation of bacteria in dental plaque. It is characterized by progressive destruction of the tooth‐supporting gum tissue and bone (Gasner & Schure, 2023). It has been reported that *P. intermedia* is positively related to clinical measures of chronic periodontitis, especially increasing pocket depth (Socransky et al., 1998), clinical attachment loss (Dahlen et al., 2014), and bleeding on probing (Joshi et al., 2014). Furthermore, *P. intermedia* has been detected in extraoral sites, such as NOMA (cancrum oris) lesions (Enwonwu et al., 2000) and bacterial tracheitis in children (Brook et al., 1997). Notably, it is the only periodontal pathogen known to induce severe bacteremic pneumococcal pneumonia, accompanied by enhanced pneumococcal adhesion to lower airway cells (Nagaoka et al., 2014). As a pathogen, several adhesins (AdpC/F) (Iyer et al., 2010; Sengupta et al., 2014), cysteine proteinase interpain A (Mallorqui‐Fernandez et al., 2008), and lipopolysaccharide (LPS) (Hashimoto et al., 2003) have been identified as virulence factors in *P. intermedia*. Nonetheless, little is known about their virulence mechanisms.

To date, no protein glycosylation system has been characterized in *P. intermedia*. Nevertheless, multiple genes encoding glycosyltransferases (GTs) involved in the biosynthesis of cell walls and LPS were found in *P. intermedia*, which varied among different strains (Kwack et al., 2022). Concerning the relationship between glycoconjugates, such as glycoproteins, LPS, and peptidoglycans (Lukose et al., 2017) with periodontal disease, genetic elements in *P. intermedia* isolates from diseased sites were shown to have more unique virulence factors associated with glycoconjugate synthesis than those from healthy sites (Zhang et al., 2017). These findings highlight a potential adaptation strategy of *P. intermedia* to employ GTs for glycoconjugate syntheses in response to dynamic oral environments during the transition from healthy to diseased states. It is also conceivable that genetic variations in GTs could enable the synthesis of species‐specific glycans and glycoconjugates, ultimately influencing the degree of virulence (Kwack et al., 2022). With respect to immunogenicity, an immunobiologically active glycoprotein isolated from *P. intermedia* was established to activate immune cells from mice and gingival fibroblasts from humans via cytokine‐inducing activity (Iki et al., 1997). This glycoprotein is devoid of fatty acids, and its activity can withstand heat inactivation at 100°C for 1 h, and treatment with proteases, while susceptible to periodate treatment (Iki et al., 1997). This suggests that this particular glycoprotein is not endotoxic and that its activity is attributed to the carbohydrate instead of the protein moiety. It is also plausible that this glycoprotein is a constituent of the capsular polysaccharide complex unique in black‐pigmented bacteria such as *Porphyromonas gingivalis*, *P. intermedia*, and related species (Sugawara et al., 2001). Nevertheless, as a periodontal pathogen, it is reasonably speculated that glycosylation in *P. intermedia* might modulate the antigen exposure or host immune response, ultimately facilitating immune evasion. Overall, these studies provide evidence for the existence of protein glycosylation in *P. intermedia*.

While the type IX secretion system (T9SS) was recently studied in *P. intermedia* for the first time (Naito et al., 2022), it has been well‐studied and postulated in *P. gingivalis* that T9SS cargo proteins are secreted through the T9SS across the outer membrane, where their C‐terminal domains (CTDs) are cleaved and their new C‐termini are conjugated to LPS, enabling their attachment to the cell surface (Veith, Glew, et al., 2022). As the cargo protein is amide‐linked to the sugar residues of the LPS (Veith et al., 2020), this process of LPS modification can also be considered a form of protein glycosylation. Utilizing genetic engineering methods, the T9SS was demonstrated to be essential for black pigmentation, hemagglutination, biofilm formation, and the functioning of cell surface virulence factors in *P. intermedia* (Naito et al., 2022). Interestingly, *P. intermedia* is the only species observed to have short CTDs with many containing only 50–60 residues compared to more than 70 residues in other species examined (Veith et al., 2013). In the same study, more than 10 cargo proteins of *P. intermedia* were substantially elevated in MW consistent with LPS modification however the modification sites and attached sugars are yet to be determined.

**Figure 1:**
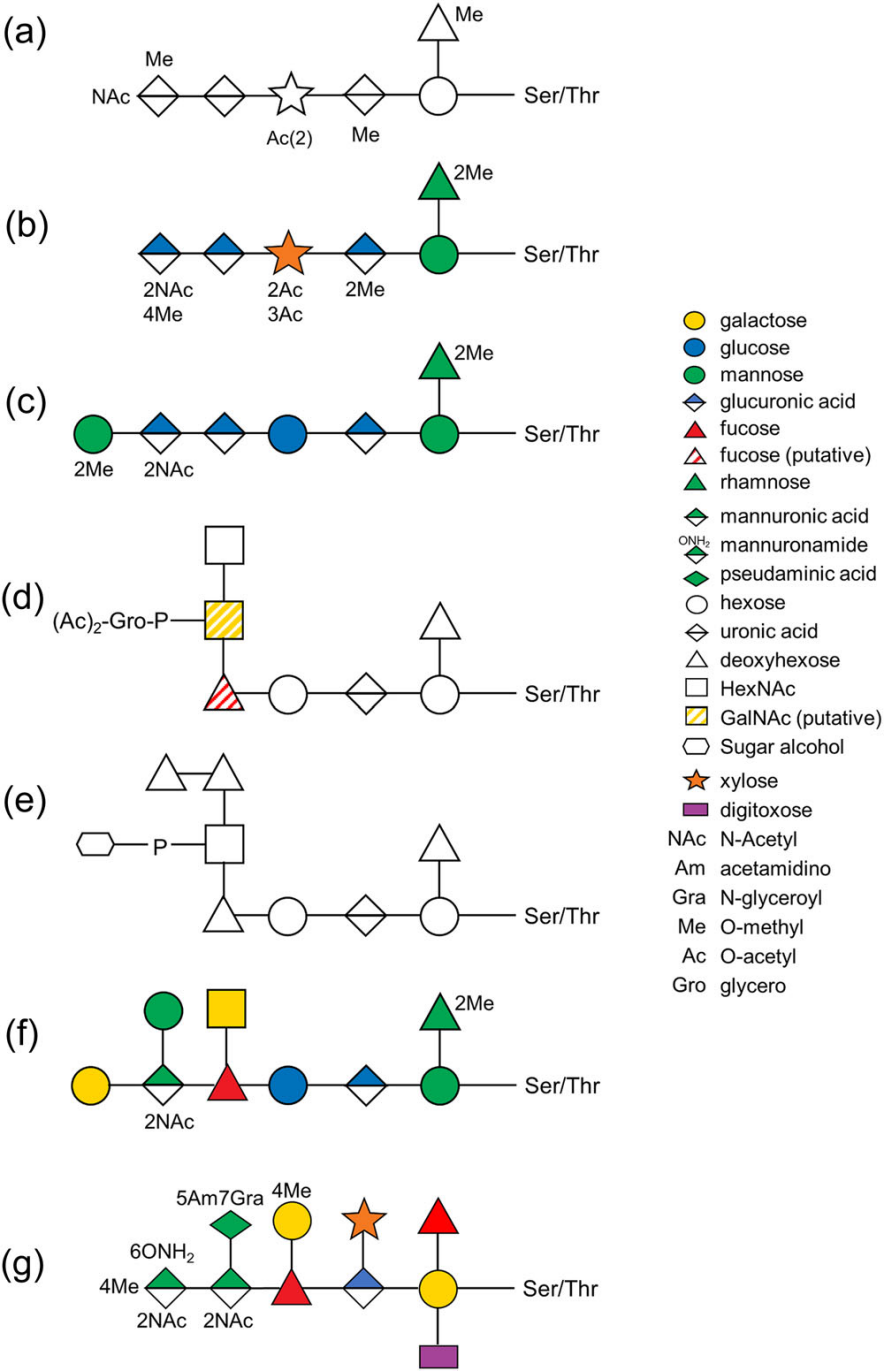
Comparison of proposed *O*‐glycan structures among *Bacteroidota* species, including (a) *Flavobacterium johnsoniae* (Veith et al., 2023), (b) *Flavobacterium columnare* (Vinogradov et al., 2003), (c) *Elizabethkingia meningoseptica* (Reinhold et al., 1995), (d) *Porphyromonas gingivalis* (Veith, Shoji, et al., 2022), (e) *Prevotella intermedia* (this study), (f) *Bacteroides fragilis* (Tomek et al., 2021), and (g) *Tannerella forsythia* (Tomek et al., 2021; Veith et al., 2021). Sugar symbols are based on Symbol Nomenclature for Glycans (Neelamegham et al., 2019). Hatched sugar symbols indicate the predicted isomeric form of the sugar.

The general *O*‐glycosylation system of the Bacteroidota phylum was first described in *Bacteroides fragilis* by Fletcher et al. (2009). By examining the glycoprotein candidates from all extracytoplasmic compartments, they experimentally confirmed the three amino acid glycosylation motif D(S/T)(A/I/L/M/T/V) in *B. fragilis*, where the glycan is *O*‐linked to the Ser or Thr residue in the second position (Fletcher et al., 2011). This *O*‐glycosylation system is now considered to be conserved across the whole Bacteroidota phylum (Coyne et al., 2013). Upon elucidation of the *O*‐glycan structure in *Elizabethkingia meningoseptica* (formerly called *Flavobacterium meningosepticum*) (Reinhold et al., 1995), *Flavobacterium columnare* (Vinogradov et al., 2003), *Tannerella forsythia* (Posch et al., 2011; Tomek et al., 2021; Veith et al., 2021), *B. fragilis* (Posch et al., 2013; Tomek et al., 2021), *P. gingivalis* (Veith, Shoji, et al., 2022), and *Flavobacterium johnsoniae* (Veith et al., 2023), it is evident that the glycan consists of a common core glycan and a more variable outer glycan (Figure 1). Despite only the first 3–4 sugars in the core glycan being similar in these species, it was suggested that the core glycan share at least a common glycan epitope, which is likely an *O*‐linked mannose or other hexose (Coyne et al., 2013). Conversely, the outer glycan is shown to be species‐specific (Coyne et al., 2013). In terms of the three amino acid motif of *O*‐glycosylation, it is conserved within numerous glycoproteins of *Bacteroidota* species and is recently extended to D(S/T)(A/I/L/M/T/V/S/C/G/F) based on the glycoproteomics studies of *T. forsythia* (Veith et al., 2021) and *P. gingivalis* (Veith, Shoji, et al., 2022). Moreover, the biosynthesis pathway of the outer glycans has been partially elucidated for *T. forsythia*, *B. fragilis*, and *P. gingivalis* (Coyne et al., 2013; Tomek et al., 2018, 2021; Veith, Shoji, et al., 2022). Among these, *T. forsythia* is the best studied with the roles of five GTs being well‐documented (Tomek et al., 2018). Furthermore, *O*‐glycosylation in *T. forsythia* was shown to modulate interspecies associations in a 10‐species biofilm model, (Bloch et al., 2017) consistent with a role for surface *O*‐glycans as ligands for coadhesion interactions.

In this study, we demonstrate the presence of the *O*‐glycosylation system in *P. intermedia* for the first time by characterizing its *O*‐glycoproteome with mass spectrometry, and where possible, relate it to ultrastructural features of *P. intermedia* cells and outer membrane vesicles (OMVs) as revealed by cryo‐electron tomography (cryoET). We identify 443 putative *O*‐glycosylation sites within 224 glycoproteins, determine the *O*‐glycan sequence, and extend the list of *O*‐glycosylation motifs.

## 2 MATERIALS AND METHODS

### 2.1 Growth of *P. intermedia*

*P. intermedia* ATCC 25611 was grown in 25 g/L brain‐heart infusion (BHI) broth and 30 g/L tryptic soy broth, supplemented with 5 µg/mL hemin, 1 µg/mL vitamin K, and 0.5 g/L cysteine under anaerobic conditions (80% N_2_, 10% CO_2_, and 10% H_2_) at 37°C for 24 h (Veith et al., 2013).

### 2.2 CryoET sample preparation and imaging

R2/2 carbon‐coated 200 mesh copper Quantifoil grids (Quantifoil Micro Tools) were first glow‐discharged for 30 s to enhance their hydrophilicity. One microliter of bovine serum albumin‐treated 10‐nm colloidal gold solution was added to 4 μL of cell suspension (OD_650_ 1.5), which was then pipetted onto a Quantifoil grid inside a Vitrobot chamber (FEI) with 100% humidity. The extra fluid was blotted off using a Whatman filter paper and the grid was plunge‐frozen in liquid ethane. The grid was imaged using a Titan Krios G4 cryoEM, operating at 300 kV acceleration voltage and equipped with a Gatan energy filter and a K3 Summit direct detector. Tilt‐series were acquired using Tomography 5 software version 5.14 (Thermo Fisher Scientific) with a tilt range of −51° to 51° in 3° increments. Data were collected with a total dose of 120 e^−^/Å^2^, a defocus of around −8 μm, and a pixel size of 3.39 Å. Three‐dimensional reconstructions of tilt‐series were performed using the IMOD software package (Kremer et al., 1996). To increase interpretability, missing‐wedge correction was done on the tomograms using IsoNet version 0.2 (Liu et al., 2022).

### 2.3 Cell fractionation

*P. intermedia* cells were harvested by centrifugation at 8000*g* for 20 min at 4°C and OMVs were pelleted from the filtered cell‐free culture fluid by ultracentrifugation at 175,000*g* for 15 h at 4°C. Cell pellets were washed with phosphate‐buffered saline (PBS) and re‐pelleted by centrifugation at 8000*g* for 20 min at 4°C. After resuspension in an acid salt buffer (ASB, 300 mM NaCl, 50 mM sodium acetate, pH 5.3), cells were lysed by two passages through a precooled Avestin EmulsiFlex C3 high‐pressure homogenizer (Avestin) at ~25,000 psi. Unlysed cells were removed by centrifugation at 8000*g* for 20 min at 4°C. The supernatant was then ultracentrifuged at 100,000*g* for 30 min at 4°C to pellet and separate the membrane fraction from the soluble fraction. The membrane fraction was resuspended in ASB with sonication using a CPX 750 ultrasonic homogenizer (Cole Parmer) fitted with a 6.5 mm tapered microtip to generate a suspension of fine particles. The amplitude was set to 19% and the pulse to 1 s on, 2 s off for a total of 15 min. A portion of the whole membrane fraction was pelleted again by centrifugation at 42,000*g* for 20 min at 4°C. Another portion of the membrane fraction was treated with 1% TRITON X‐100 detergent and mixed by rotation for 0.5 h at room temperature. The extracted membrane fraction [or inner membrane fraction (IM)] was the supernatant retained after centrifugation at 42,000*g* for 20 min at 4°C. Portions of the soluble fraction and OMV sample was precipitated with 13% trichloroacetic acid (TCA) by centrifugation at 15,000*g* for 20 min at 4°C. The pellets were washed with ice‐cold acetone and centrifuged again.

### 2.4 Partial deglycosylation

Portions of membrane fraction, precipitated soluble fraction and OMV sample were resuspended in 50% acetonitrile–0.1% aqueous trifluoroacetic acid (TFA), transferred to reaction vials and freeze‐dried thoroughly overnight. Deglycosylation was performed following the protocol provided by the manufacturer of the PROzyme/Glyko Glycofree chemical deglycosylation kit (GKK‐500) (ProZyme & Inc, n.d.) as previously described (Veith et al., 2020). All the following steps were conducted in the fume hood due to the highly volatile and corrosive nature of trifluoromethanesulfonic acid (TFMS). Briefly, samples were placed in an ethanol/dry ice bath and 125 μL of TFMS/toluene mixture (90% TFMS, 10% anhydrous toluene) was slowly added using the predried glass syringes. Reaction vials were placed in a freezer for 10 min at −20°C. After the first 5 min of deglycosylation reaction, the vials were briefly shaken to assist with the melting of the contents and the subsequent solvation of glycoproteins. The samples were then slowly neutralized with 375 μL (3 volumes) of pyridine/methanol/water solution at a ratio of 3:1:1 in the ethanol/dry ice bath. After 5 min of neutralization on dry ice and 15 min on wet ice, samples were transferred to microcentrifuge tubes and 1 mL (8 vol) of 50 mM ammonium bicarbonate (NH₄HCO₃) was then added. Deglycosylated polypeptides in samples were recovered by precipitation with 13% TCA and washed with ice‐cold acetone.

### 2.5 Sodium dodecyl sulfate–polyacrylamide gel electrophoresis (SDS‐PAGE) and in‐gel digestion

**Figure 2:**
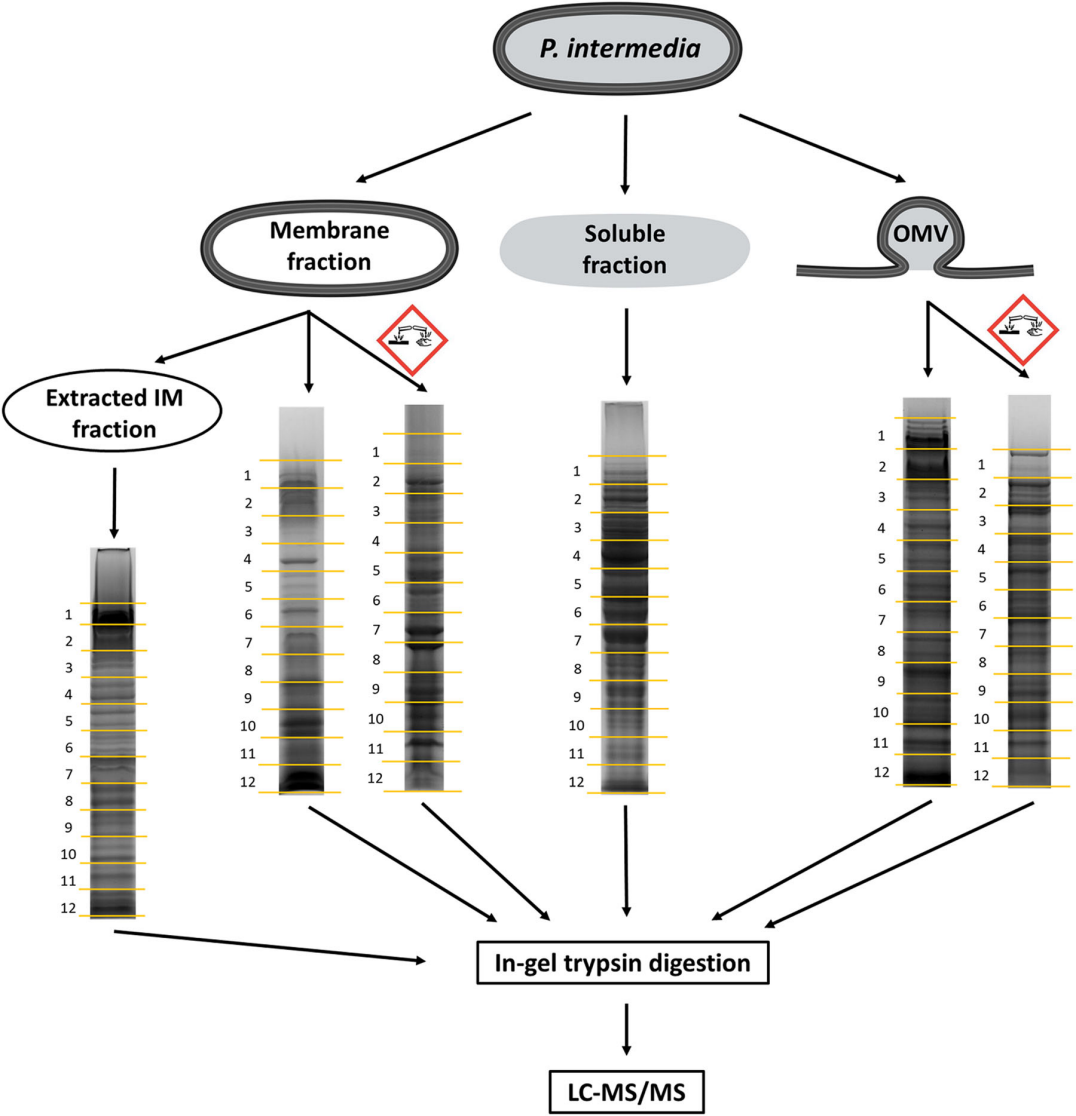
Outline of sample preparation. Harvested *Prevotella intermedia* cells were initially divided into membrane, soluble, and outer membrane vesicles (OMV) fractions. A portion of the membrane fraction was extracted with detergent to enrich inner membrane proteins. Another fraction of the membrane sample and a fraction of the OMV sample were partially deglycosylated with trifluoromethanesulfonic acid (indicated by the corrosive hazard symbol). All six samples were subjected to sodium dodecyl sulfate–polyacrylamide gel electrophoresis, excision into 12 equal gel segments, and in‐gel digestion with trypsin and liquid chromatography coupled to tandem mass spectrometry.

Untreated samples of whole membrane, soluble, extracted membrane and OMVs, and deglycosylated samples were dissolved in 1× NuPAGE LDS sample buffer and 50 mM dithiothreitol to denature proteins. After sonication and heating, all samples were separated by reducing SDS‐PAGE and fractionated into 12 gel segments respectively (Figure 2). The segments were digested with trypsin in the gel as described previously (Gorasia et al., 2015) and extracted once with 0.1% aqueous trifluoroacetic acid (TFA) and once with 30% acetonitrile–0.1% aqueous TFA, both for 15 min, in an ultrasonication bath. Extracts were combined, evaporated in a vacuum centrifuge, and dissolved in 2% acetonitrile‐0.1% aqueous TFA for MS analysis.

### 2.6 Mass spectrometry

LC‐MS/MS experiments were conducted on a Dionex Ultimate 3000 UHPLC interfaced with an Orbitrap Fusion Lumos Tribrid mass spectrometer (Thermo Fisher Scientific) as previously described (Veith, Shoji, et al., 2022), with the following modifications. For the analysis of intact glycopeptides, peptides were eluted using a linear gradient of 2%–40% ACN over 85 min to obtain the high‐energy collisional dissociation (HCD) and collision‐induced dissociation (CID) spectra. A stepped field asymmetric ion mobility spectrometry (FAIMS) method was employed alternating between the compensation voltages (CVs) of −25 and −45 V. Specific glycan fragment ions (204.09, 244.03, 448.12 *m*/*z*) were used to trigger the additional CID scan, stepped collision energy HCD scan and electron transfer dissociation (ETD) or electron‐transfer/higher‐energy collision dissociation (EThcD) scans (all in the orbitrap) with previously described scanning parameters (Veith, Shoji, et al., 2022). The ETD and EThcD parameters were NCE 15%, a maximal injection time of 250 ms with an AGC of 500%, and a resolution of 30,000 using the extended mass range setting. MS3 spectra were acquired for the sample with the highest abundance of glycopeptides (gel segment #8 of the membrane fraction) to identify the sequence of the unknown glycan portion. A stepped FAIMS method was utilized again, with the following modifications. An inclusion mass list of the previously identified glycopeptide ions with delta masses (Δmasses) of ~1531 Da was used to trigger the MS2‐level HCD spectra. Additional CID and HCD scans were conducted on the 448.12 *m/z* and 594.18 fragment ions in two separate experiments to obtain the MS3‐level spectra.

For the analysis of acid‐cleaved glycopeptides, membrane, and OMV samples were eluted using a 60‐min gradient of approximately 2%–32% ACN. A FAIMS method alternating between −25 and −45 V was utilized, and only HCD spectra were collected. For the acquisition of CID spectra, an inclusion mass list of the previously identified glycopeptide ions was used to trigger the CID scans in separate experiments. Electron transfer dissociation (ETD) and electron‐transfer/higher‐energy collision dissociation (EThcD) scans were acquired in a separate experiment using similar inclusion lists but restricting the charge state to 3–8 (both singly and doubly charged ions were excluded).

### 2.7 Peptide identification

Proteins and peptides were identified by searching against the *Prevotella intermedia* ATCC 25611 sequence database of 2156 protein sequences downloaded from UniProt Proteomes (Proteome ID = UP000187195). All searches were performed using trypsin and other parameters were as follows. Maximum missed cleavages = 2, peptide mass tolerance = 10 ppm, fragment mass tolerance = 0.04 Da, fixed modification = cysteine carbamidomethyl, and variable modifications = methionine oxidation.

Initially, the raw MS data were searched with Byonic v4.6 (Protein Metrics) using the wildcard parameters with a Δmass between 200 and 2000 Da (Roushan et al., 2021). The result files were exported via Byonic Viewer v4.6. Once the most abundant glycoform was identified, the data were searched again using 1531.48 (S, T) as an additional variable modification. A glycopeptide was considered identified and included in Table S2 when it contained the glycosylation motif and was detected more than once, with a Byonic score >200 and –Log10 (*P*) > 1. The same data was also searched using Mascot v2.8.2 (Matrix Science), with scores over 30 included. The false discovery rate (FDR) using the Mascot decoy for intact samples was 1.17% for all peptides, and 0% for glycopeptides.

For deglycosylated samples, an error‐tolerant Mascot search was conducted with the error‐tolerant modifications edited to include only the list of putative fragments specific to the *P. intermedia* glycan in addition to oxidation (M) set as a variable modification as usual. Only glycopeptides with a Mascot score >30 were considered identified unless they were supporting an already identified site. Due to incompatibility between decoy mode and error‐tolerant mode, the FDR was determined only for the most abundant modifications (Hex2‐HexA and dHex‐Hex). The FDR was considered insignificant as glycopeptides (score >30) with either modification were absent in the decoy database search result.

Most glycosylation sites were supported by more than one peptide sequence or from multiple glycoforms (i.e., after deglycosylation). Manual validation of glycosylated peptides was performed for glycosylation sites that were only identified from one sequence/glycoform where the Byonic score was <300 or the Mascot score was less than 35. To pass validation, the MS^2^ spectra needed to exhibit (i) the 448.12 *m/z* glycan fragment ion; (ii) a prominent Y_0_ peak followed by peaks at +338, +500, and +646 Da corresponding to the first four sugars of the glycan; (iii) a convincing series of b‐ions and y‐ions consistent with positive peptide identification.

### 2.8 Protein localization

Proteins were localized by bioinformatic approaches as follows. IM proteins and OM proteins were predicted based on the presence of transmembrane IM α‐helices (TMH) and OM β‐barrels respectively using the DeepTMHMM server v1.0.20 (https://dtu.biolib.com/DeepTMHMM/) (Hallgren, 2022). A BLASTp search (NCBI) was performed to identify the *P. intermedia* T9SS cargo proteins with Uniprot accession numbers equivalent to the T9SS cargo proteins with another type of accession numbers obtained from our previous report (Veith et al., 2013). These proteins were considered as cell surface proteins along with newly predicted cargo proteins. Lipoproteins located in the cell membrane were predicted using SignalP‐6.0 (https://services.healthtech.dtu.dk/services/SignalP-6.0/) (Teufel et al., 2022) and validated by the presence of signal peptides from the DeepTMHMM search. Proteins predicted to be periplasmic were selected from the remaining proteins when signal peptides were detected by both SignalP‐6.0 and DeepTMHMM. The remaining unassigned proteins lacked signal peptides and were therefore predicted to be cytoplasmic. Alternatively, these proteins might utilize atypical secretion pathways for their export and could also be assigned as proteins of “uncertain” location.

### 2.9 Glycan nomenclature

The nomenclature employed for depicting and abbreviating sugars was adopted from the Symbol Nomenclature for Glycans (SNFG) (https://www.ncbi.nlm.nih.gov/glycans/snfg.html) (Neelamegham et al., 2019).

## 3 RESULTS

### 3.1 CryoET analysis of *P. intermedia* cells and OMVs

**Figure 3:**
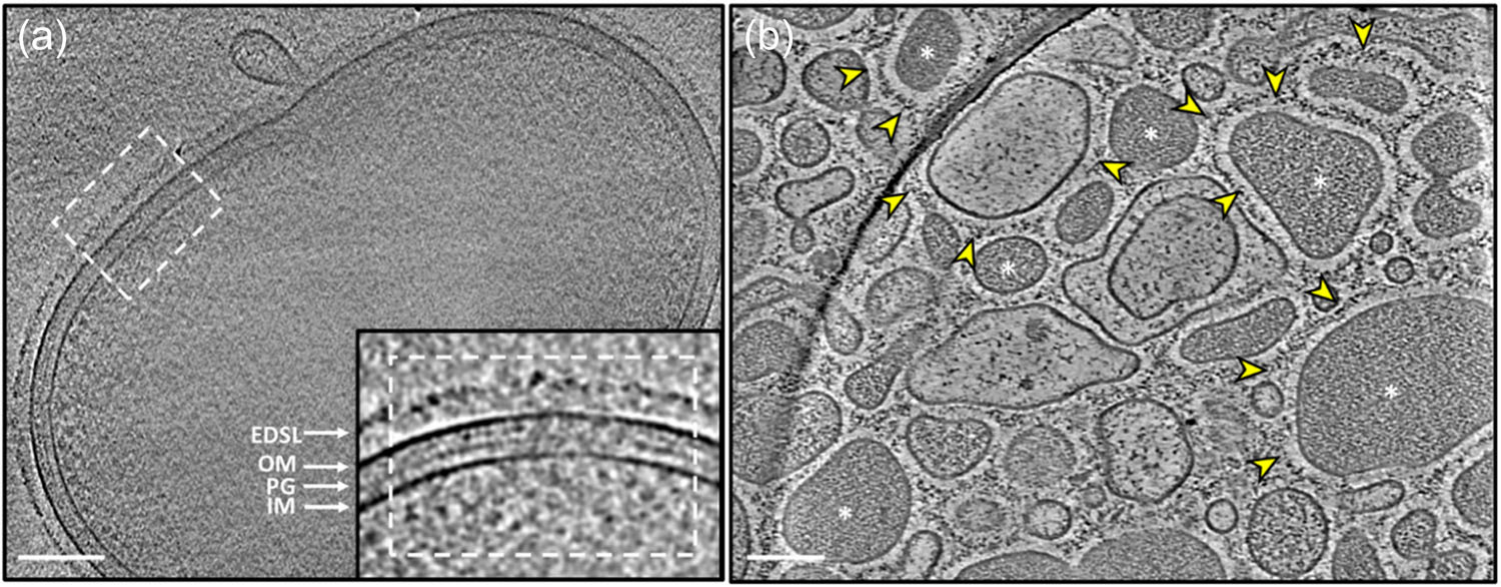
CryoET of *Prevotella intermedia* cells and outer membrane vesicles (OMVs). (a) Representative tomographic slice showing ultrastructural details of *P. intermedia*. Inset: magnified image revealing structural details of the cell envelope, including the inner membrane (IM), the peptidoglycan layer (PG), the outer membrane (OM), and an electron‐dense surface layer (EDSL). (b) Tomographic slice of purified OMVs showing the presence of a thick EDSL (yellow arrowhead) surrounding the membrane of different‐sized vesicles. There are translucent and electron‐dense (white star) OMVs. The scale bar is 100 nm.

The cryoET analysis of *P. intermedia* revealed cells are of coccobacillus shape measuring approximately 1.5 μm in length and 0.9 μm in diameter. Similar to *P. gingivalis*, it also possessed an electron‐dense surface layer (EDSL) outside the outer membrane (OM) that plays a role in bacterial interactions (Figure 3a). Cryotomograms of purified OMVs revealed that *P. intermedia* generates a wide range of OMVs, varying in size (~0.01 to 0.6 μm wide), shape, and in their appearance (Figure 3b). Notably, these OMVs were also enveloped by the EDSL, showcasing a unique aspect of their composition and arrangement. Interestingly, some OMVs displayed electron‐dense interiors, indicative of the presence of proteinaceous substances while others appeared relatively empty (Figure 3b). This observation provides valuable insights into the potential functional diversity of OMVs produced by *P. intermedia*.

### 3.2 Overall identification of the glycoproteome

**Figure 4:**
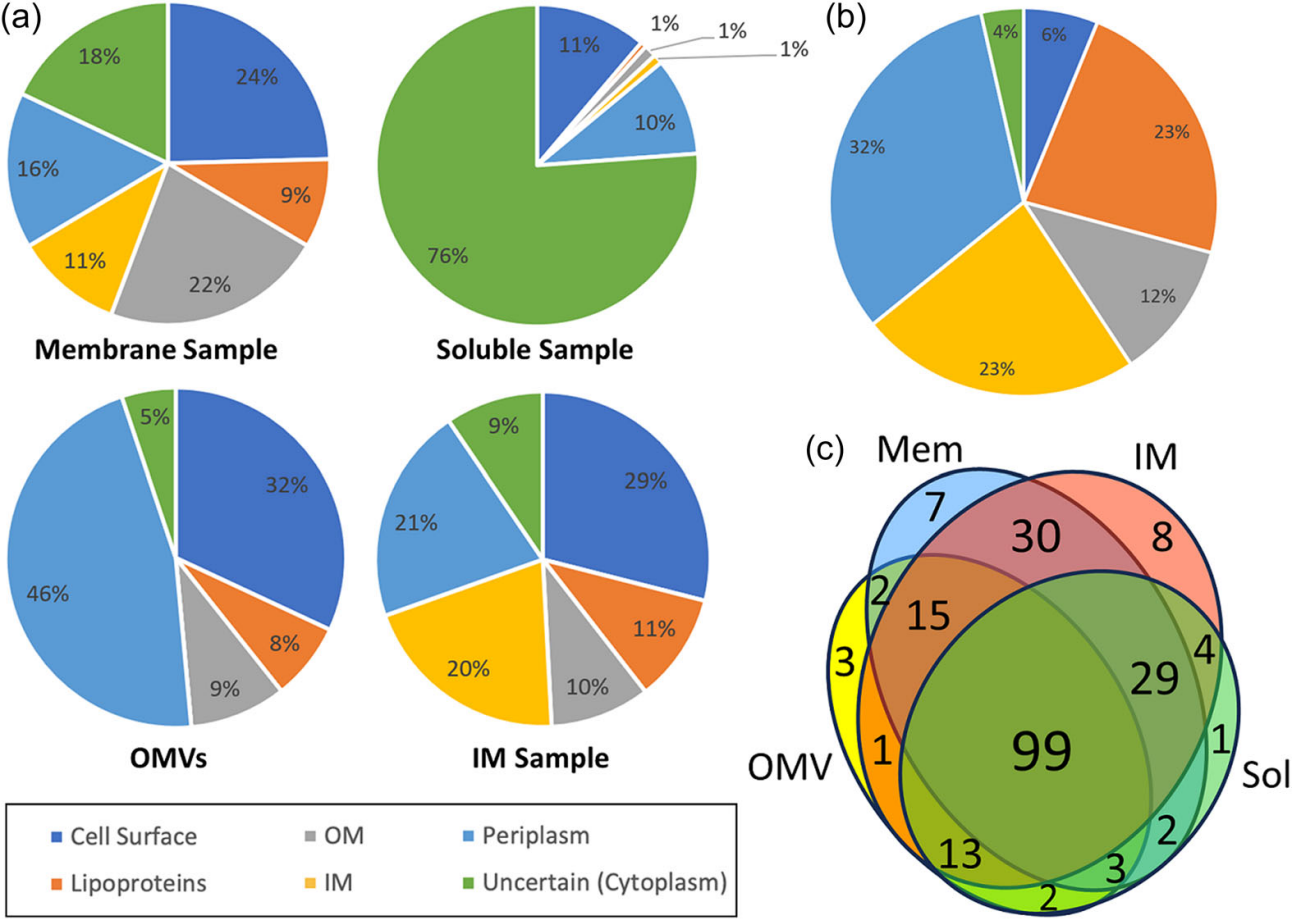
Fractionation efficiency and predicted subcellular localization. (a, b) The predicted subcellular localization of proteins is provided with the proportion of proteins being estimated by summing the Mascot scores for that locale. The assignment of protein localization was based on prediction tools including DeepTMHMM and SignalP. Proteins of uncertain localization that lack signal peptides were assigned as cytoplasmic. T9SS cargo proteins were considered cell surface proteins. Lipoproteins are associated with the inner membrane (IM) or outer membrane (OM). (a) According to all identified proteins across each sample fraction (membrane, soluble, outer membrane vesicles, and IM). Only the top 100 proteins with the highest Mascot protein scores in each fraction are included. The localization of all proteins is listed in Table S1. (b) Glycoproteins across all fractions. Only glycoproteins containing the putative glycosylation motif are shown. The predicted localization of individual glycoproteins is listed in Table S3. (c) Venn diagram analysis showing overlap of glycoprotein identification between fractions. Only strongly identified abundant glycoproteins (Mascot score >200) were used for this analysis.

*P. intermedia* cultures were fractionated into soluble, membrane, extracted IM, and OMV fractions. Partial deglycosylation was only performed on the membrane and OMV fractions. Intact or deglycosylated samples were subjected to SDS‐PAGE, excision into 12 gel segments, in‐gel digestion with trypsin, and LC‐MS/MS analysis (Figure 2). The raw MS/MS data were searched using both Byonic and Mascot. Table S1 lists the complete set of 1655 unique proteins detected from all fractions in *P. intermedia*. Overall, 574 glycopeptides within 224 glycoproteins containing 443 unique *O*‐glycosylation sites were identified (Tables S2 and S3). These glycoproteins were found to be modified by a unique glycan moiety (see below). The fractionation was designed to allow the enrichment of exported proteins and hence increase the number of glycoproteins identified. The fractionation efficiency was assessed by predicting the localization of each protein identified (Table S1) and then plotting the proportion of each locale represented in the various fractions (Figure 4a). The membrane sample proved the best source for OM proteins; the OMVs were the best source of periplasmic and surface proteins; and the extracted membrane sample was the best source of IM proteins and lipoproteins (Figure 4a). The 224 identified glycoproteins were predicted to localize predominantly in the periplasm (73 glycoproteins), IM (53), or found to be lipoproteins (52) (Figure 4b and Table S3). Twenty‐six proteins were predicted to be located in the OM, and six proteins were cytoplasmic or of uncertain localization. Only 14 were the predicted T9SS cargo proteins, possessing a total of 22 identified *O*‐glycosylation sites. Venn diagram analysis shows the relative importance of each fraction for the identification of glycoproteins (Figure 4c). While all fractions were useful for the identification of glycoproteins, the extracted membrane fraction (“IM”) and total membrane fraction enabled the highest numbers of glycoproteins to be identified. The most heavily glycosylated proteins, characterized by the highest number of identified glycosylation sites were BWX39_03000 (OstA‐like_N domain‐containing protein), BWX39_01155 (Big_5 domain‐containing protein) and BWX39_07550 (kinase), with 10, 7, and 7 identified glycosylation sites, respectively (Table S3).

### 3.3 Determination of glycoforms

**Figure 5:**
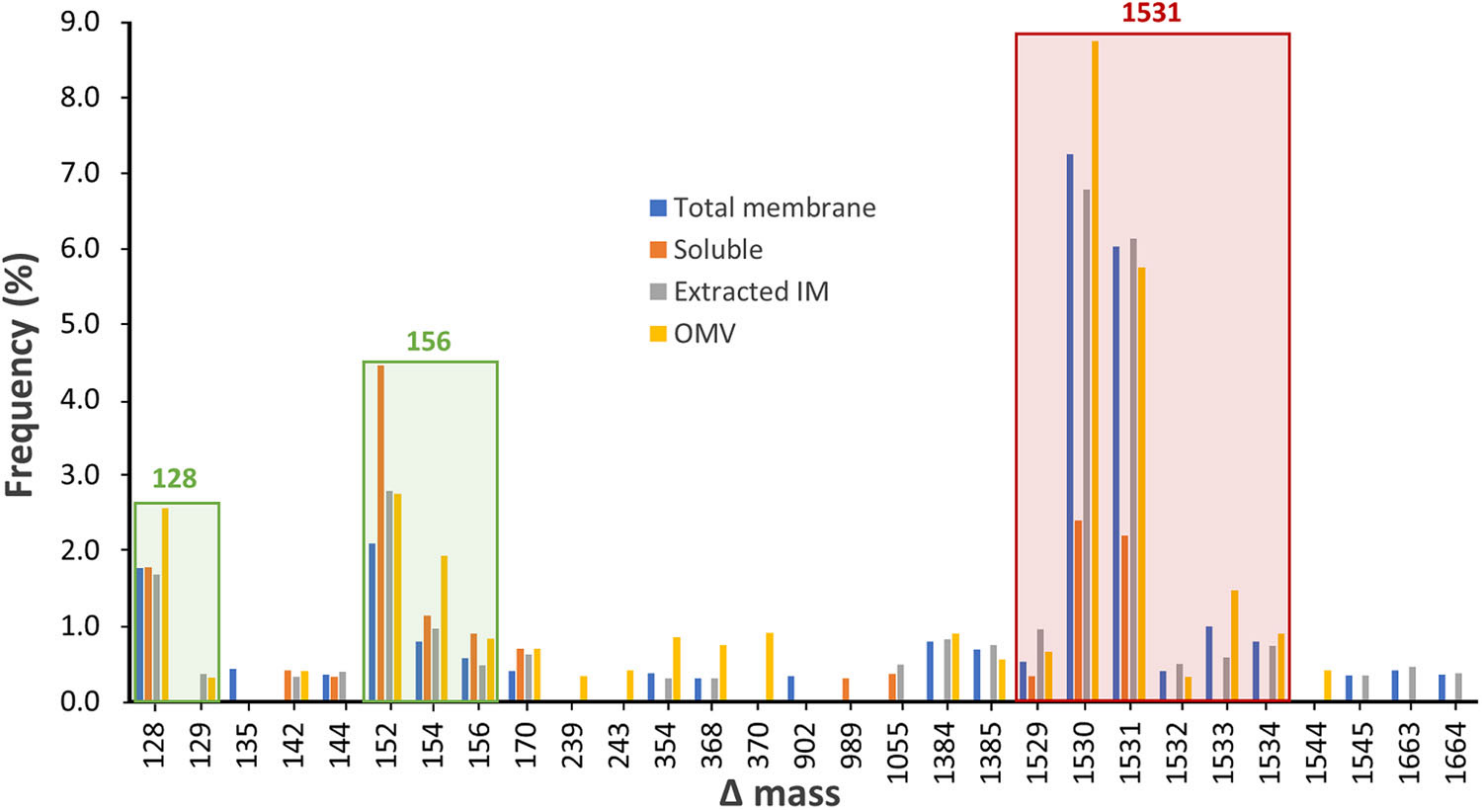
Frequency of glycoforms detected by Byonic. The intact glycopeptides of membrane, soluble, extracted inner membrane, and outer membrane vesicles fractions were searched by Byonic using the wildcard setting. The three most abundant Δmass values were grouped into clusters in red and green and the corrected integer Δmass values are indicated above each cluster. Only Δmass values with a frequency >0.3% among all modified peptides are displayed. 128 and 156 Da modifications represent additional Lys and Arg residues (missed cleavages) at the peptide N‐ or C‐termini.

To identify the glycan sequences linked to the glycosylated proteins, the initial step was to determine the masses of potential glycoforms. The MS/MS data obtained from the intact samples were searched using Byonic with a wildcard setting, which allowed the identification of peptides modified with any delta mass (Δmass) value. A plot representing the frequency of Δmass values among the identified peptides exhibited a predominant high Δmass cluster of potential glycopeptides (Figure 5). The 1531‐Da cluster highlighted in red, differing by 1‐Da units, was found to be the most frequently observed in all fractions (Figure 5). Some of the other abundant clusters with low Δmass values could be attributed to Lys (128 Da) and Arg (156 Da) due to the misidentification of peptides containing two adjacent tryptic cleavage sites (Figure 5).

Through manual inspection of the MS1 spectra corresponding to peptides within the ~1531‐Da Δmass cluster, a total of 136 monoisotopic masses were recorded and the majority of the calculated Δmass fell within the 1531‐Da integer potentially representing a unique glycoform (Table S4). The clustering of these Δmass values could be the result of the inaccurate assignment of the monoisotopic peak by Byonic. Other less frequent values within the range of 1529–1534 Da corresponded to poorly defined isotopic envelopes and were deemed outliers. The accurate mass of the *O*‐glycan was determined to be 1531.48 Da by averaging the Δmass values around 1531 Da only (Table S4).

The identified peptides with a potential modification of ~1531 Da were also examined through their corresponding MS/MS spectra. They were preliminarily identified as glycopeptides based on the presence of oxonium ions of sugar fragments in the MS2‐level spectra, such as HexNAc (*N*‐acetylhexosamine) at *m/z* 204.09, Hex (hexose) at *m/z* 163.06, and dHex (deoxyhexose) at *m/z* 147.07. The putative *O*‐glycosylation motif D(S/T)(A/I/L/M/T/V/S/C/G/F/N) was present in almost all of these identified peptides, which further supported their identity as glycopeptides. The modification of ~1531.48 Da was therefore conceived to be the dominant *O*‐glycan in *P. intermedia*.

The data were then searched again in Byonic using 1531.48 Da, specific to Ser or Thr, as another variable modification. A greater number of glycosylated peptides were identified, which encompassed nearly all the peptides with a Δmass of ~1531 Da from the wildcard searches. In all fractions, a total of 512 different peptides were identified, out of which 40 lacked the putative D(S/T) X motif (Table S5). Through manual inspection of the HCD spectra, the presence or absence of the 204.09 *m/z* HexNAc oxonium ion was recorded and found to be present in 32 of these spectra. This suggests that most are indeed glycopeptides, however correct assignment of the peptide sequence was still in doubt. Since peptides lacking the motif were more likely to be false positives, only the top three having a Byonic score >400 were considered positively identified and included in the main tables (Table S2 and S3). These three sequences displayed potential glycosylation sites at ETI, ESV, and ESV respectively (Table S5).

### 3.4 Identification of the glycan sequence

**Figure 6:**
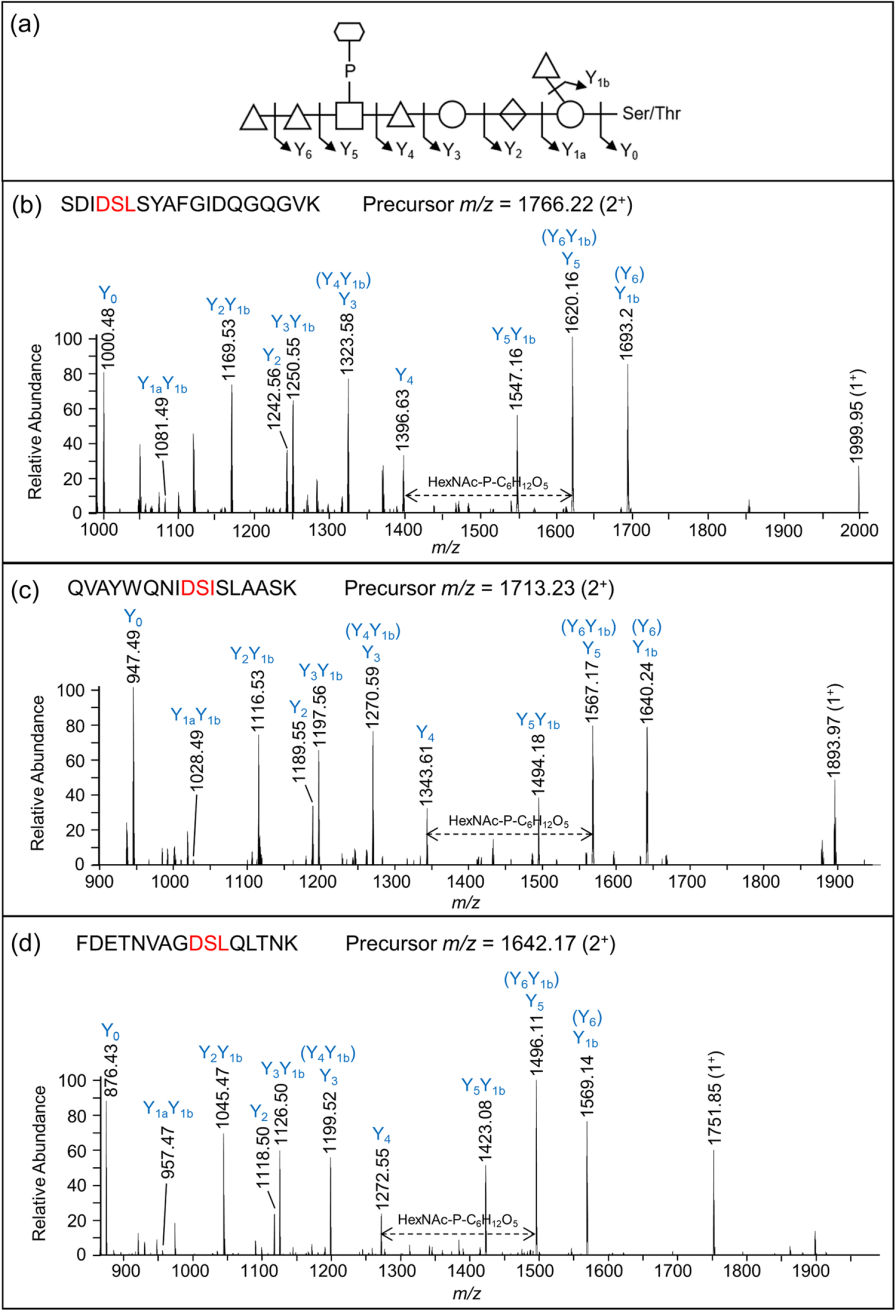
Collision‐induced dissociation (CID) spectra of glycopeptides modified with the 1531‐Da glycoform. (a) Proposed sequence of the *O*‐glycan showing the fragmentation scheme. (b–d) CID spectra of three glycopeptides. The identified glycopeptide sequences are shown with the putative *O*‐glycosylation motifs highlighted in red. The precursor *m/z* values are indicated for each glycopeptide. Y_0_ corresponds to the unmodified peptides. The three glycopeptides shown originated from the membrane sample (gel segments #10, 9, and 11, respectively). The CID spectra were triggered by the presence of the glycan fragment ion at 448.12 *m/z*. The labeled ions are doubly charged unless specified as 1^+^. Note that some ions have two labels due to these fragments having the same mass. For example, the Y_3_ ion cannot be differentiated from the Y_4_Y_1b_ ion.

**Figure 7:**
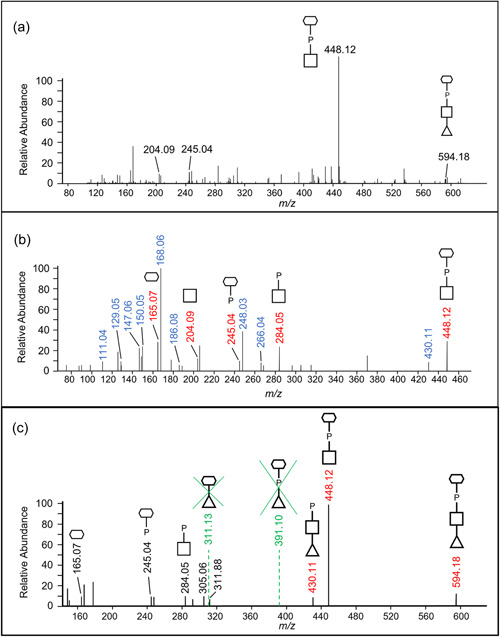
High‐energy collisional dissociation (HCD) spectra to characterize the 447 Da moiety. (a) A representative MS2‐level HCD spectrum showing common peaks in the low mass region. The peaks at 204.09, 245.04, and 448.12 *m/z* represent the predicted 203.08‐Da HexNAc, the unknown 244.03‐Da portion, and the 447.11‐Da moiety, respectively. These labeled ions in the low‐mass region were common across all inspected spectra of glycopeptides. (b) The MS3‐level spectrum of the 448.12 *m/z* ion. The ions shown in blue represent losses of water from the ions of interest (Table 1). (c) MS3 spectrum of the 594.18 *m/z* ion to confirm the position of C_6_H_12_O_5_ and HexNAc. The fragment ions of interest that correspond to the hypothesized moieties are highlighted in red. All fragment ions are singly charged. the signed sequence of the glycan.

**Figure 8:**
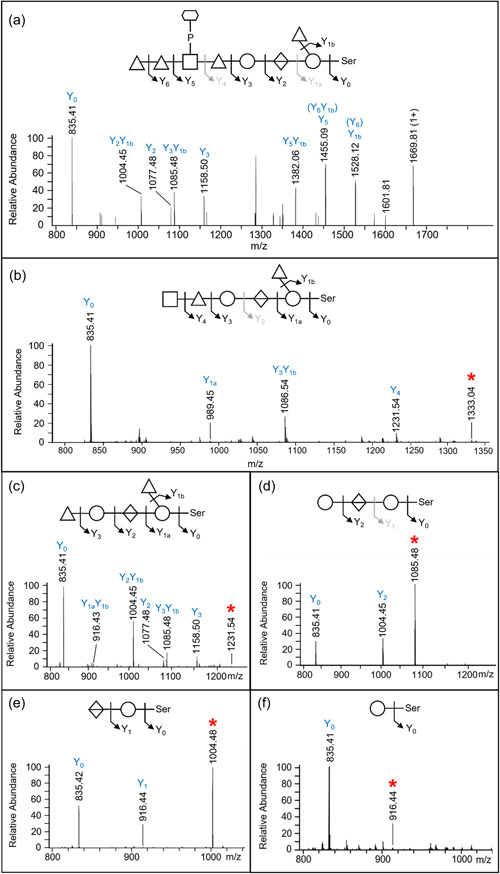
Collision‐induced dissociation (CID) spectra of partially deglycosylated glycopeptides. The glycans are derived from the peptide DGAVYFLQANDSTLR within the protein BWX39_07850 (copper resistance protein NlpE). (a) represents the full‐length sequence of the glycan. (b–f) matches the Δmass value of each acid‐cleaved glycan, which is 995 (792 + HexNAc), 792, 500, 338, or 162 Da, respectively. The peaks corresponding to precursor ions are indicated by red asterisks (*). Notations in grey indicate the positions where the cleavage is absent in that particular spectrum.

The proposed sequence of the 1531.48‐Da *O*‐glycan is dHex‐dHex‐HexNAc(HPO_3_‐C_6_H_12_O_5_)‐dHex‐Hex‐HexA‐Hex(dHex) (Figure 1e). Its elucidation is described in detail below, but in outline, it was determined by: (i) analyzing the CID spectra corresponding to glycopeptides with a Δmass of 1531.48 Da (Figure 6); (ii) the HexNAc(HPO_3_‐C_6_H_12_O_5_) portion was determined based on additional HCD spectra (Figure 7); and (iii) the whole sequence was confirmed by partial deglycosylation of glycopeptides with TFMS (Figure 8).

The CID spectra of distinct glycopeptides with an accurate Δmass of 1531.48 Da exhibited consistent fragmentation patterns allowing the first five sugars (from reducing end, right to left) to be deduced as hexose, deoxyhexose side branch, hexuronate, hexose, and deoxyhexose (Figure 6). The 6th group between the Y_4_ and Y_5_ ions had a mass of 447.11 Da which was later assigned to HexNAc(HPO_3_‐C_6_H_12_O_5_). The last two sugars were assigned to deoxyhexoses (Figure 6). Rarely were any peaks observed to help dissect the structure of the 447.11 Da moiety, however, in Figure 6a, a peak at *m*/z 1538.14 was consistent with our assignment of HexNAc‐P followed by C_6_H_12_O_5_.

To identify the unknown 447‐Da component, the HCD spectra of identified glycopeptides were manually inspected. The common peaks across all glycopeptide HCD spectra should represent the sugar residues of the *O*‐glycan, whereas the variable peaks should correspond to the amino acid residues of different peptides. The peak representing the 448.12 *m/z* ion displayed the highest intensity across all inspected spectra and accurately matched the 447 Da component plus a proton for ionization (Figure 7a). The peaks at 204.09 and 245.04 *m/z* were also consistently present in all spectra examined and appeared to be complementary fragments of the 447 Da species (Figure 7a). After matching the accurate masses to their molecular formula, it was hypothesized that the unknown moiety between Y_4_ and Y_5_ consisted of a HexNAc residue (203.08 Da) and a C_6_H_13_O_8_P moiety (244.03 Da).

To further examine the composition of the 447‐Da moiety, MS3‐level HCD scans were conducted on the 448.12 *m/z* ion. The peaks of interest, 204.086 *m/z*, and 245.04 *m/z*, were present in the HCD spectra confirming their origin in the 447 Da moiety (Figure 7b). The accurate masses and respective molecular formula of the fragment ions are listed in Table 1. Many ions could be assigned to the loss of water molecules (–18 Da), a common feature of sugar fragmentation. Except for those fragment ions assigned to a loss of H_2_O, the peak at 165.0748 *m/z* exhibited relatively high intensity and matched best to C_6_H_12_O_5_ (Figure 7b). A search for known structures that correspond to this accurate mass using Metlin resulted in the best matches to sugar alcohols such as mannitol, sorbitol, galactitol, or iditol. The presence of extra fragment ions due to H_2_O losses (147.0646, 129.0542, and 111.0435 *m/z*) supported the assignment of the 165 *m*/*z* component to a sugar (Figure 7b and Table 1). Finally, the remaining moiety could be accurately attributed to a phosphate residue (HPO_3_), which is commonly observed in complex glycans of bacteria and yeast. Collectively, the data indicate that the 447.11‐Da portion is composed of a HexNAc, a phosphate group, and a C_6_H_12_O_5_ sugar alcohol residue. The presence of peaks at 245.0414 *m/z* (HPO_3_ + C_6_H_12_O_5_) and 284.0518 *m/z* (HPO_3_ + HexNAc), as well as the lack of a peak at ~368 *m/z* (HexNAc + C_6_H_12_O_5_), indicated that the phosphate is positioned in the middle (HexNAc‐HPO_3_‐C_6_H_12_O_5_).

**Table 1:** Accurate mass data from orbitrap MS3 of the 448.12 *m/z* ion.

Observed *m/z* (*z* = 1)	Molecular formula (neutral charge)^a^	Error (ppm)^b^
448.1211	C_14_H_26_NO_13_P (HexNAc‐HPO_3_‐C_6_H_12_O_5_)	0.79
430.1113	C_14_H_24_NO_12_P	0.96
284.0518	C_8_H_14_NO_8_P (HexNAc‐HPO_3_)	4.15
266.0404	C_8_H_12_NO_7_P	7.57
248.0313	C_8_H_10_NO_6_P	2.22
245.0414	C_6_H_13_O_8_P (HPO_3_‐C_6_H_12_O_5_)	2.78
204.0855	C_8_H_13_NO_5_ (HexNAc)	5.63
186.0754	C_8_H_11_NO_4_	3.68
168.0648	C_8_H_9_NO_3_	4.28
165.0748	C_6_H_12_O_5_ (C_6_H_12_O_5_)	5.92
150.0545	C_8_H_7_NO_2_	3.03
147.0646	C_6_H_10_O_4_	3.98
129.0542	C_6_H_8_O_3_	3.26
111.0435	C_6_H_6_O_2_	0.56

^a^ The molecular formulae corresponding to the accurate masses in the first column were deduced using ChemCal. The assigned compounds are indicated in blue. All other ions potentially represent losses of water from the ions of interest in blue.

^b^ The mass error is the relative difference between the observed mass of an ion and its calculated mass based on the assigned molecular formula.

Given that the 447.11‐Da moiety is linked to a dHex on both sides in the glycan, the conclusion as to whether HexNAc or C_6_H_14_O_6_ is linked to dHex could be drawn by examining the MS3 HCD scan of the 594.18 *m/z* ion (HexNAc + HPO_3_ + C_6_H_12_O_5_ + dHex). As shown in Figure 7c, the presence of the 430.11 *m/z* peak was consistent with the dHex linked to the HexNAc although it could also arise by the loss of H_2_O from the 448.12 *m/z* ion. Nonetheless, the absence of the 391.10 *m/z* (dHex + C_6_H_12_O_5_ + HPO_3_) and 311.13 *m/z* (dHex + C_6_H_12_O_5_) peaks supported that the dHex residue was not linked to the C_6_H_12_O_5_ residue (Figure 7c).

To confirm the glycan sequence identified in the intact glycopeptides, the MS/MS data of acid‐cleaved glycopeptides were initially subjected to the Byonic wildcard search. The frequent Δmass values observed were consistent with the cleavages expected from the assigned glycan sequence. Mascot searches were then conducted involving defined modifications corresponding to the different portions of the 1531.48‐Da glycan, including 162, 308, 338, 484, 500, 646, 792, 849, 995, 1093, 1239, and 1385 Da. The most frequent Δmass values were at 500.14 and 338.08 Da, which accurately matched the Hex‐HexA‐Hex and Hex‐HexA portions respectively (Table 2). This finding indicates the strong preference of TFMS to cleave at the dHex residues. Very few peptides were found to be cleaved around the HexNAc residue (Table 2). The glycopeptides identified in the partially deglycosylated sample exhibited a significant overlap with those modified with intact glycans (Table S2).

**Table 2:** Frequency of partially deglycosylated glycans and intact glycans.

Δmass	162	308	338	484	500	646	792	849	995	1093	1239	1385	1531
Frequency	48	4	191	2	343	49	2	2	3	1	2	6	40

*Note*: Δmass values between 162 and 1385 Da indicate the acid‐cleaved glycan portions. 1531 Da represents the intact *O*‐glycan.

To characterize the sequence of acid‐cleaved glycans, CID scans were performed. Figure 8 illustrates the same peptide, DGAVYFLQANDSTLR modified with uncleaved glycan (Figure 8a) and progressively truncated glycan (Figure 8b–f). The spectrum shown in Figure 8b confirmed the location of HexNAc as the 6th sugar in the sequence, which together with the data shown in Figure 7 completes the sequence determination of the outer glycan. The remaining CID spectra showed a sequential loss of a HexNAc, two dHex residues, a Hex, and a HexA confirming the assigned sequence of the glycan.

### 3.5 Localization of glycosylation sites

**Figure 9:**
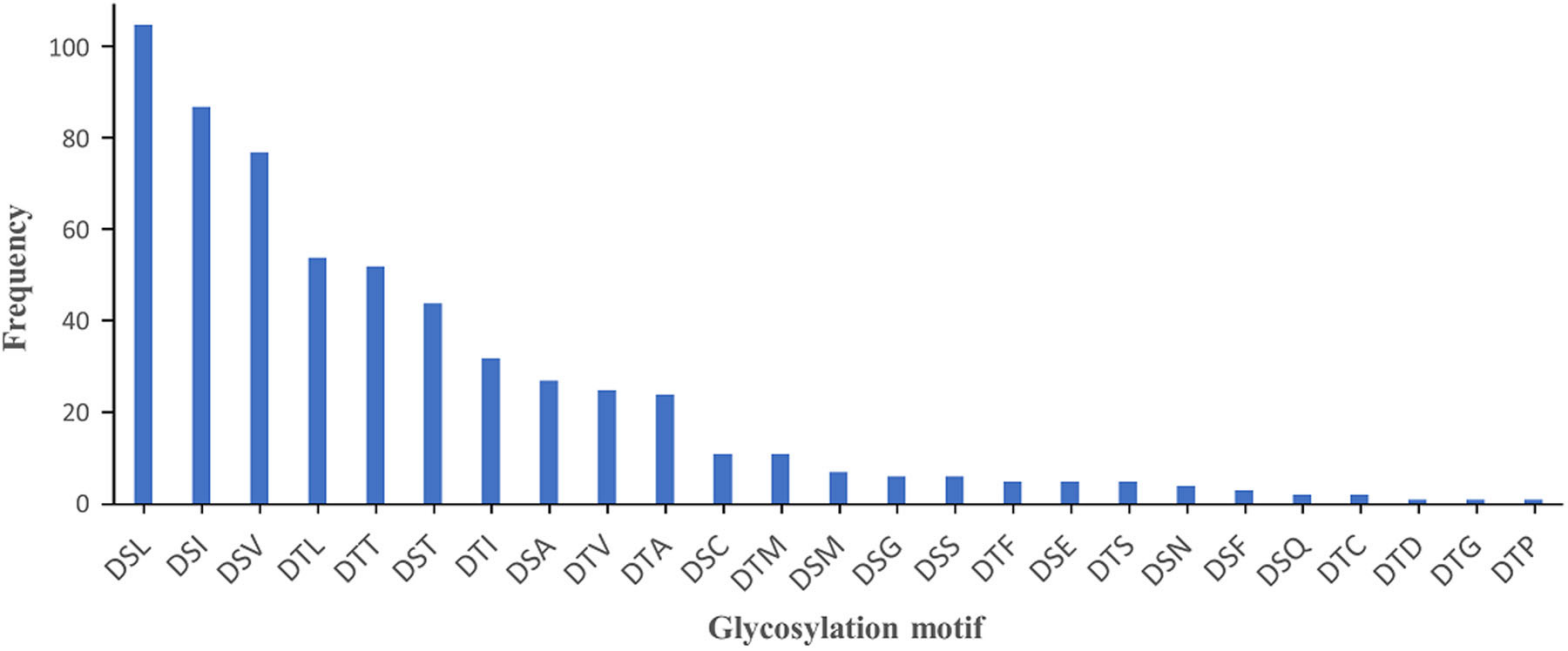
Frequency of *O*‐glycosylation motifs identified in *Prevotella intermedia*. The number of glycopeptides (frequency) possessing each site ranged from 2 to 105 as shown. All except four motifsbe long to the putative Bacteroidota *O*‐glycosylation motif D(S/T) (A/I/L/V/M/T/S/G/F/C/N). The four motifs uniquely identified in *P. intermedia were DS(E/Q) and DT(D/P)*. Other newly identified motifs that were present with another putative motif in a single sequence were excluded.

Overall, 25 distinct *O*‐glycosylation motifs were identified in *P. intermedia* (Figure 9 and Table S2). The total number of identified motifs is different from the number of peptides identified since a few sequences have more than one possible motif. *P. intermedia* had a strong preference for DS (L/I/V) residues and a relatively low preference for D(S/T) (G/F/C/S). The DSN motif was uniquely observed in *F. johnsoniae* (Veith et al., 2023) and *P. intermedia*. Interestingly, 4 new motifs, DS(E/Q) and DT(D/P) were identified in *P. intermedia* (Figure 9 and Table S2). The Δmod score quantifies the difference in Byonic scores of the top matching site and the second‐best scoring site. It indicates the confidence of the glycosylation site assignment in a peptide spectrum match. In this study, Ser or Thr was manually defined as the modification site, and therefore, the Δmod score was only available when more than one Ser/Thr residue was present in a glycopeptide. For glycopeptides with a Δmod score >15, 23 out of 26 glycans in intact peptides, and 34 out of 47 glycans in acid‐cleaved peptides were assigned to the putative D(S/T) X motifs (Table S2). When the ΔMod score exceeded 30, all of the glycosylation sites were correctly assigned by Byonic (Table S2), providing robust evidence for glycosylation at the putative motifs.

**Figure 10:**
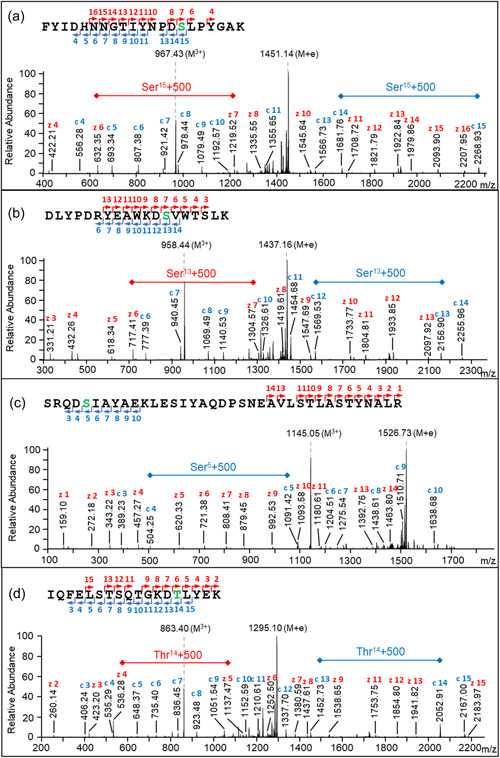
Electron transfer dissociation (ETD) or electron‐transfer/higher‐energy collision dissociation (EThcD) spectra showing localization of glycosylation sites. The peptides are (a) FYIDHNNGTIYNPDSLPYGAK, (b) DLYPDRYEAWKDSVWTSLK, (c) SRQDSIAYAEKLESIYAQDPSNEAVLSTLASTYNALR, and (d) IQFELSTSQTGKDTLYEK. Deglycosylated samples were analyzed by ETD or EThcD by use of an inclusion list to fragment the most appropriate precursor ions (see Section Materials and Methods). The residual glycan of 500 Da at Ser or Thr highlighted in green indicates the glycosylation site in each of the four different sequences. All the c‐ions and z‐ions are 1^+^ charged and labeled in blue and red, respectively. The letter M indicates the precursor ion in each spectrum.

To localize the exact *O*‐glycosylation sites of some peptides, ETD or EThcD fragmentation was applied to the deglycosylated samples. It enables fragmentation along the peptide backbone, thereby keeping the glycan moiety linked to the amino acid, directly showing the site of glycan attachment (Catalina et al., 2007). Four ETD or EThcD spectra are provided in Figure 10a–d, showing the 500‐Da modification precisely located at the Ser or Thr residues within the DSL, DSV, DSI, and DTL motifs, respectively, which were the four most abundant *O*‐glycosylation motifs found in *P. intermedia* (Figure 9). Series of c‐ and z‐ions demonstrated the presence of the modification at the expected motif instead of other potential sites (Figure 10). For example, as shown in Figure 10b, the 500‐Da glycan fragment accounts for the large gaps between z6 and z7 ions (red), and between c12 and c13 ions (blue), indicating the site of modification at the Ser residue (green) in the middle. All the other ions correspond to each amino acid sequentially.

## 4 DISCUSSION

This is the first comprehensive report of *O*‐glycosylation in *P. intermedia* and any *Prevotella* species. Overall, 574 glycopeptides within 224 glycoproteins containing 443 unique *O*‐glycosylation sites were experimentally identified (Tables S2 and S3). A previous study predicted, 389 candidate glycoproteins in *P. intermedia* strain 17, exclusively based on the presence of the *O*‐glycosylation motif D(S/T) (A/L/V/I/M/T) in proteome sequences (Coyne et al., 2013). Given that different strains within each species share a similar number of glycoproteins, (Coyne et al., 2013) the number of glycoproteins (224) identified in *P. intermedia* ATCC 25611 in this study is approximately 58% of the number of predicted candidate glycoproteins (389). Fewer glycosylation sites and glycoproteins were identified in our previous studies of *P. gingivalis* (257 *O*‐glycosylation sites within 145 glycoproteins, 40% of candidates) (Veith, Shoji, et al., 2022), *T. forsythia* (312 sites in 145 glycoproteins, 26% of candidates (Veith et al., 2021), and *F. johnsoniae* (325 sites in 226 glycoproteins, 22% of candidates) (Veith et al., 2023). The higher numbers for *P. intermedia* may reflect better fractionation and optimized protocols.

Based on a series of MS/MS analyses, the *O*‐glycan sequence was determined to be a 1531.48‐Da dHex‐dHex‐HexNAc(HPO_3_‐C_6_H_12_O_5_)‐dHex‐Hex‐HexA‐Hex(dHex) (Figure 1e). As could be expected, the first three sugars (from the right), a hexose, a deoxyhexose, and a hexuronate are similar across all seven species whose *O*‐glycan sequences or structures have been determined (Figure 1). In particular, the third sugar, HexA is a glucuronic acid in all known cases. This is consistent with the cross‐reactivity of an antibody against the “core glycan” of *B. fragilis* with glycoproteins in all tested species from different classes of Bacteroidota (Coyne et al., 2013 *)*. Although the “core glycan” was defined as the first two sugars of the *O*‐glycan in that study, the purity of the “core glycan” was not demonstrated (Coyne et al., 2013). Furthermore, the GT responsible for transferring the third sugar hexuronate remains unidentified (Coyne et al., 2013; Tomek et al., 2018; Veith, Shoji, et al., 2022). Consequently, the antibodies generated against this core glycan may potentially recognize additional sugars such as the well‐conserved hexuronate (Figure 1). Beyond the HexA, the sequences become more divergent. *T. forsythia* generates the most complex and distinct *O*‐glycan, featured by the presence of side branches on every sugar in the main chain (Figure 1g). The *O*‐glycan in *P. intermedia* closely resembles that in *P. gingivalis* and *B. fragilis*, with the first six sugars being generically the same (Figure 1d–f). Notably, the sugar alcohol moiety present in the side branch of the O‐glycan in *P. intermedia* is novel among O‐glycans identified in Bacteroidota bacteria to date. This sugar appears to be more commonly present in yeast glycans, as evidenced by a study demonstrating the prevalence of sugar alcohols as constituents in the most abundant *O*‐glycans in *Pichia pastoris* (Trimble et al., 2004).

The general *O*‐glycosylation system was first described in *B. fragilis*, in which numerous proteins are glycosylated at a conserved three amino‐acid motif D(S/T) (A/I/L/M/T/V), with the glycan attached to the Ser or Thr residue (Fletcher et al., 2009). Subsequent glycoproteomic studies on *T. forsythia* and *P. gingivalis* extended the list to D(S/T) (A/I/L/M/T/V/S/C/G/F) (Veith et al., 2021; Veith, Shoji, et al., 2022). Further comparison of site preferences for *O*‐glycosylation among *T. forsythia*, *P. gingivalis*, and *F. johnsoniae*, revealed that the DT(C/G/M) motif was not observed in *F. johnsoniae* while the DSN motif was uniquely detected in *F. johnsoniae* (Veith et al., 2023). In *P. intermedia*, *O*‐glycosylation exhibits a strong preference for the DS(L/I/V) motif and DSN is also present. Additionally, among all newly identified D(S/T)X motifs, DS(E/Q) and DT(D/P) were found to be glycosylated in *P. intermedia*. Therefore, the list of *O*‐glycosylation motifs could now be extended to D(S/T) (A/I/L/M/T/V/S/C/G/F/N/E/Q/D/P), encompassing all amino acids except for R, H, K, W, and Y in the third residue position (Figure 9).

Surprisingly, some peptides were detected with a 1531.48‐Da Δmass but did not contain the D(S/T)X motif (Table S5). Of these identifications, three were of sufficient quality to show glycosylation at two new motifs, ETI and ESV, both exhibiting a conservative substitution of Asp with a Glu. It is well‐established that the functioning of *O*‐OTase depends on various factors, including the presence of specific amino acid residues or structural motifs in the target sequence and the recognition of specific glycosylation signals (Chen et al., 2016; Musumeci et al., 2013; Rini et al., 2022), these unusual glycosylation sites reflect that *O*‐OTase of *P. intermedia* appears to have compromised specificity compared to the *O*‐OTase of other species.

Previous studies in *B. fragilis* suggested that *O*‐glycosylation functions stabilize glycoproteins, as evidenced by the apparent instability of the unglycosylated protein (Fletcher et al., 2009). Additionally, in *T. forsythia*, *O*‐glycosylation contributes to bacterial virulence, in which mutants with truncated glycans elicited varied behaviors in terms of biofilm formation and immunogenicity (Bloch et al., 2017). Further investigations are necessary to elucidate the roles of *O*‐glycosylation in *P. intermedia*.

Previous proteomic analysis of *P. intermedia* compared the subcellular localization of proteins in planktonic and biofilm states (Karched et al., 2022). However, OMVs in *P. intermedia* have yet to be explored. It was only after prolonging the ultracentrifugation time overnight (15 h) and increasing the ultracentrifugation speed (175,000*g*) that sufficient OMVs were finally obtained, suggesting a relatively low abundance of OMVs in *P. intermedia*. As shown in Figure 4a, most of the protein content (54%) identified in the OMV fraction was of periplasmic origin, followed by T9SS cargo proteins located on the vesicle surface. Interestingly, this OMV protein composition differs significantly from that observed in *P. gingivalis* (Veith et al., 2018) and *T. forsythia* (Veith et al., 2015), where the OM and cell surface proteins made up over 95% of OMV protein. This result can be explained by the cryoET analysis. *P. intermedia* OMVs varied considerably in size, but many were more than 200 nm wide (Figure 3) whereas OMVs from *P. gingivalis* (Veith et al., 2014) and *T. forsythia* (Veith et al., 2015) were on average much smaller. Not only did *P. intermedia* OMVs have larger lumens with the capacity for more periplasmic proteins, but a sizeable proportion of the OMVs displayed electron‐dense lumens, suggesting a high density of periplasmic proteins. The two distinct populations of OMVs with electron‐dense or electron‐transparent lumens suggest two different mechanisms for their biogenesis, one where periplasmic proteins appear to be excluded from the OMVs, likely similar to the mechanisms in *P. gingivalis* and *T. forsythia*, and a unique mechanism where periplasmic proteins are included.

The *O*‐glycoproteins identified in *P. intermedia* are predominantly localized to the periplasm, IM, or associated with the membrane as lipoproteins in the periplasm (Figure 4b). Two types of *O*‐glycoproteins, peptidyl‐prolyl cis–trans isomerases and tetratricopeptide repeat (TPR) domain proteins were frequently observed (Table S3). Their homologs in *P. gingivalis* were found to be essential for its growth and colonisation (Kishi et al., 2012), suggesting similar roles in *P. intermedia*. Consistent with the finding that *O*‐glycosylation occurs outside the cytoplasm, *O*‐glycoproteins predicted to localize in the IM are mostly those with large periplasmic domains, such as gliding motility protein GldM (PorM), fusion protein SecF, signal peptidases, cell division proteins, penicillin‐binding proteins, DUF490 domain‐containing proteins, and YjgP/YjgQ family permeases (Table S3). Fewer glycoproteins localized to the OM, where the majority of glycosylation sites were found in T9SS component proteins, Sov and PorF, as well as the Omp85 (BamA) proteins responsible for assembling β‐barrel Omps (Table S3). Biofilms of deglycosylated BamA mutants in *P. gingivalis* were more susceptible to inhibition by the antibody targeting BamA, (Nakao et al., 2008) suggesting its glycosylation provides a survival advantage during biofilm formation. Six *O*‐glycoproteins were predicted to localize in the cytoplasm due to the absence of a predicted signal peptide (Table S3). Further research is required to determine if these are exported via an atypical mechanism.

The T9SS was recently studied in *P. intermedia* for the first time, demonstrating that a functional T9SS is essential for black pigmentation, hemagglutination, biofilm formation, and the functioning of cell surface virulence factors (Naito et al., 2022). Fourteen T9SS cargo proteins, including key virulence factors such as adhesins, internalins, peptidases, hemin‐binding protein, and leucine‐rich repeat domain‐containing protein, (Karched et al., 2022) were found to be *O*‐glycosylated in *P. intermedia* at a total of 22 sites (Table S3). As LPS‐modification of cargo proteins is also a form of glycosylation, these 14 T9SS cargo proteins likely undergo two types of glycosylation, while many of the remaining T9SS cargo proteins might only be glycosylated at the new C‐terminus by the linking sugars in LPS. The LPS‐modified cargo proteins are understood to be located in the EDSL layer surrounding both cells and OMVs (Figure 3). This level of O‐glycosylation in T9SS cargo is in‐between that observed for *T. forsythia* where 120 glycosylation sites were identified in 18 cargo proteins, (Veith et al., 2021) and *P. gingivalis* where only 7 sites from 5 low abundance cargo were identified (Veith, Shoji, et al., 2022). In our *P. gingivalis* study, we concluded that O‐glycosylation of surface proteins was under negative selection, but this is less clear in *P. intermedia*. Regarding the 19 T9SS protein components identified in *P. intermedia* so far (Naito et al., 2022), nine were found to be *O*‐glycosylated, including PorE/F/K/M/N/W/Y/U and Sov with a total of 26 glycosylation sites (Table S3). This emphasizes the importance of *O*‐glycosylation in bacterial virulence, particularly in relation to the secretion of virulence factors and other critical factors for bacterial coaggregation and biofilm formation. However, whether the absence of *O*‐glycan in these T9SS components would compromise the proper functioning of the T9SS remains unknown.

In summary, this is the first study to report the O‐glycoproteome and demonstrate a functional *O*‐glycosylation system in *P. intermedia*. We showed that *P. intermedia* utilizes a novel *O*‐glycan containing a rare sugar alcohol that predominantly targets proteins located in the periplasm. The *O*‐glycoproteome extended to OMVs which harbored an unusually large number of periplasmic proteins and glycoproteins which could be seen in cryoET images as having electron‐dense lumens.

## AUTHOR CONTRIBUTIONS

**Xi Ye**: Investigation; writing—original draft; visualization. **Bindusmita Paul**: Investigation; writing—original draft; visualization. **Joyce Mo**: Investigation. **Eric C. Reynolds**: Funding acquisition; resources; supervision. **Debnath Ghosal**: Funding acquisition; resources; supervision; writing—review and editing. **Paul D. Veith**: Conceptualization; writing—review and editing; supervision; project administration; methodology.

## CONFLICTS OF INTEREST STATEMENT

None declared.

## ETHICS STATEMENT

None required.

## Supporting information


**Table S1.** Proteome data and predicted localization of all identified proteins.
**Table S2.** Identified glycopeptides and their glycosylation sites and motifs.
**Table S3.** Identified glycoproteins and their predicted localization.
**Table S4.** Calculation of accurate Δmass values of O‐glycopeptides.
**Table S5.** Byonic searches with the 1531.48‐Da modification for intact glycopeptides.

## Data Availability

The mass spectrometry proteomics data have been deposited to the ProteomeXchange Consortium via the PRIDE partner repository (Perez‐Riverol et al., 2022) with the data set identifier PXD047143: https://doi.org/10.6019/PXD047143.
